# 

**Published:** 1894-02

**Authors:** 


**ESTABLISHED 1855.** **SUBSCRIPTION, $2.00.**

### ATLANTA MEDICAL AND SURGICAL JOURNAL.

OLD SERIES, VOL. XXVI. NEW SERIES, VOL. X. } ATLANTA, GA., FEBRUARY, 1894. No. 12.

LUTHER B. GRANDY, M. D., MANAGING EDITOR. M. B. HUTCHINS, M. D., BUSINESS MANAGER.

